# The Growing Relevance of Immunoregulation in Pediatric Brain Tumors

**DOI:** 10.3390/cancers13225601

**Published:** 2021-11-09

**Authors:** Viktoria Melcher, Kornelius Kerl

**Affiliations:** Department of Pediatric Hematology and Oncology, University Children’s Hospital Münster, 48149 Münster, Germany

**Keywords:** pediatric brain tumors, embryonal brain tumors, atypical teratoid/rhabdoid tumor, ependymoma, high-grade glioma, low-grade glioma, medulloblastoma, therapy resistance, tumor microenvironment, immune cells, immunomodulation, immunotherapy

## Abstract

**Simple Summary:**

Pediatric brain tumors are the leading cause of childhood cancer-related deaths worldwide. Considering the dismal prognosis and the adverse effects of chemo- and radio-therapy, strategies targeting the tumor microenvironment represent a promising approach for improving the efficacy of standard and targeted molecular therapeutics. This review presents the current understanding of the juvenile innate immune system in the central nervous system and gives insights into the brain as a unique tumor site. Moreover, we outline an explorative overview of studies about the tumor microenvironment of pediatric brain tumors and its role in tumor progression and therapy resistance. We further put attention to the potential immunomodulatory effects of current therapeutic regimens. Finally, we provide a perspective regarding the present immunotherapeutic treatment options and future clinical implications of targeting the immune cells.

**Abstract:**

Pediatric brain tumors are genetically heterogeneous solid neoplasms. With a prevailing poor prognosis and widespread resistance to conventional multimodal therapy, these aggressive tumors are the leading cause of childhood cancer-related deaths worldwide. Advancement in molecular research revealed their unique genetic and epigenetic characteristics and paved the way for more defined prognostication and targeted therapeutic approaches. Furthermore, uncovering the intratumoral metrics on a single-cell level placed non-malignant cell populations such as innate immune cells into the context of tumor manifestation and progression. Targeting immune cells in pediatric brain tumors entails unique challenges but promising opportunities to improve outcome. Herein, we outline the current understanding of the role of the immune regulation in pediatric brain tumors.

## 1. Introduction

Tumors of the central nervous system (CNS) are the most common solid tumors in children and the leading cause of worldwide childhood cancer-related deaths [1]. The most prevalent pediatric brain tumors are categorized according to World Health Organization (WHO) into low-grade gliomas (LGG), high-grade gliomas (HGG), ependymomas (EPN), and embryonal brain tumors. The latter are high-grade neoplasms, originating from undifferentiated embryonic cells, and are classified into atypical teratoid/rhabdoid tumor (ATRT), medulloblastoma (MB), embryonal tumor with multilayered rosettes (ETMR), and other CNS embryonal tumors (previously named CNS primitive neuroectodermal tumor) [2]. Pediatric brain tumors have been profoundly characterized on the genomic and transcriptomic levels. Besides recurrent mutations in distinct signaling pathways (e.g., sonic hedgehog (SHH), Wnt/wingless (WNT) and mitogen-activated protein kinase (MAPK)), epigenetic processes play a crucial role in their pathogenesis [3,4,5]. Comprehensive molecular analyses paved the way for entity sub-classification, allowing for a more defined prognostication and targeted therapeutic approaches [6,7,8]. However, patients undergo intensive multimodal therapy with surgical resection, chemo- and/or radiotherapy. These treatment regimens have multiple adverse effects and often cause neurocognitive deficits [9,10].

Besides the increasing knowledge about molecular subgroups and their implications on clinical outcome in pediatric brain tumors, much less is known about the role of infiltrating immune cells in these malignancies. In contrast, in adult cancer, the discipline of cancer immunotherapy has grown largely in recent years [11]. As brain tumors in children and adults are distinct on different levels, e.g., frequency, anatomic location, as well as the pathologic, genetic and epigenetic spectrum, it might not be possible to extrapolate results from adult studies to pediatric tumor entities [12]. Pediatric cancers are generally assumed to be less immunogenic, which might be based on their low mutational burden [13,14].

However, recent efforts have shown that antitumor immune control exists in pediatric brain tumors, e.g., rhabdoid tumors and LGG showed strong T cell infiltration [15,16,17]. Moreover, several studies present prognostications of specific immune cell infiltrates on overall survival [18,19,20,21,22] (Table 1). Thus, the indications that host immunity may already impact patients’ survival in pediatric brain tumors suggests that immune profiling can add valuable information in diagnostics and assessment of immunotherapeutic therapies for individual patients.

This review aims at summarizing the current knowledge of tumor-associated immune cells in pediatric brain tumors and further considers important aspects for the implementation of immunotherapeutic targets to conventional therapy.

## 2. The (Postnatal) Brain as a Unique Tumor Site and CNS Immune Surveillance

Malignancies developing in highly organized organs such as the central nervous system (CNS) can severely impact the whole organism. In the (adult) tumor-developing brain, the stroma is initially composed of neuronal cells, astrocytes, oligodendrocytes, and microglia surrounded by a distinctive extracellular matrix (ECM) [23]. However, due to the complexity of brain development and its (context-dependent) cellular interactions already under physiological conditions, crosstalk between these cell populations and tumorigenic cells in the developing brain is barely characterized. During brain development, the generation of neurons and glial cells is precisely regulated by diverse mechanisms, including the immune system. The first immune cells that encounter the developing brain are microglia [24,25]. This section discusses how the immune system interacts with tumor cells in general and how distinct immune cells specifically contribute to tumor cell growth in the developing brain.

Generally, in the setting of tumor development, the immune system operates at multiple levels when preventing and eliminating cancerous cells while sparing healthy cells: viral infections that can induce tumors are eliminated or at least suppressed; pathogens are killed and the consequential inflammation is controlled to prevent an inflammatory environment that is susceptible for carcinogenesis; and lastly, innate immune cells recognize stressed cells or tumor-specific antigens and molecules and eradicate (pre-)tumorigenic cells. However, the fact that tumors still develop under functional immune system is nowadays explained by the term “tumor immunoediting” [26]. Immunoediting describes a concept of three phases during the suppression of tumor cells by innate and adaptive immune cells: (1) elimination, (2) equilibrium, and (3) escape. First, cells that escaped mechanisms of intrinsic tumor suppression are detected and eliminated by innate immune cells. If eradication is incomplete, tumor cells can enter the second phase, tumor dormancy, and are in a temporary equilibrium with the adaptive immune system that suppresses tumor expansion. In this dynamic phase, tumor cells can evolve (further mutations; changes in gene expression that modulate tumor-specific antigens or boost immunosuppressive mechanisms) and adjust to the immune pressure, which finally leads to a selection of resistant tumor cell clones. Thus, the tumor escapes from the antitumor immune response and progresses [27]. More recent studies revealed that innate immune cells can undergo cancer immunoediting in the absence of adaptive immunity [28].

Studies on brain tumor biology and immunology mechanisms in adults outweigh infant and juvenile tumor immunology knowledge. Immune mechanisms are predominantly studied in infectious diseases, which is plausible, considering the low incidence of pediatric (brain) cancer compared to the age-related increase in adults. Though many processes are similar in pediatric and adult immunity, there are substantial age-specific developmental changes through the transition from the fetal stage to neonatal and infant. Most of all, the immune system does not fully mature until approximately the age of 12 years. Due to that, generalizing results from adult research to children should be interpreted with caution [29]. Most notably, the fetal, neonatal and infant stages of development require immune suppression. Otherwise, conditions like the semi-allogeneic state of the mother/fetus during pregnancy or the transition from a protected environment in utero to postnatal rapidly changing environmental influences would result in systemic inflammation. This is in part realized via a tolerogenic immune response involving Treg cells as well as IL-10 production by neonatal antigen-presenting cells (APCs) and high concentrations of the immunosuppressive purine metabolite adenosine [30,31,32,33]. Thus, children are born with relatively naive T and B cells, hypofunctional innate immunity and fetal red blood cells [24].

Another aspect of the brain as a unique tumor site is the physical protection by the blood–brain barrier (BBB), a tight unity of endothelial cells, astrocytes, pericytes and microglia. This cellular cohesion protects the CNS from pathogens and prevents neurotoxicity by inflammation from peripherally infiltrating immune cells. Further, the CNS lacks classical lymphatic drainage, meaning the transport route of antigens and APCs from the inflammation site to the nearby lymph nodes [34]. Hence, it has long been believed that the brain is immune privileged. Still, the brain also has direct access to the blood system via circumventricular organs, which serve as a direct passage of cytokines [35]. Moreover, also immune cells, such as B cells, T cells, and macrophages, are observed in the meningeal, ventricular and perivascular space and choroid plexus. These cell types have crucial roles in brain function [24,34]. In CNS tumors, the permeability of the BBB is frequently altered and functionality varies between brain tumor entities [36]. In the WNT-MB subtype, tumor cells block the endothelial WNT signaling and disrupt the BBB formation, while the SHH-MB subtype showed a normal vascular phenotype. These tumor-intrinsic differences could explain why WNT-MB are vulnerable to systemic chemotherapy, which has to cross the BBB, but SHH-MB is not responding [37]. In ATRT, BBB deficits could also explain the effectiveness of chemotherapeutic treatment, resulting in improved survival of ATRT patients [38,39]. Meel et al. [40] found BBB phenotypic abnormalities in ATRT-SHH and ATRT-MYC and observed in vivo efficacy of two non-BBB penetrable drugs [40].

In the following section, we describe the divergent role of the resident and infiltrating immune cells of the CNS in tumor suppression and promotion.

### 2.1. Microglia as Brain-Resident Innate Immune Cell Populations

Microglia exhibit regenerative properties, namely myelin-debris removal, secretion of growth and neurotrophic factors, which are essential for proper remyelination of newly differentiated oligodendrocytes. Regenerative properties of microglia are associated with alternative M2-phenotypes, which secrete anti-inflammatory cytokines and growth factors. Besides that, they are the first line of innate immunity defense and, as highly motile cells, they continuously survey the brain parenchyma. This classically activated M1-phenotype is associated with antigen presentation, secretion of pro-inflammatory cytokines and reactive oxygen and nitrogen species [41]. Therefore, they are probably the first innate immune cells to interact with malignant cells. It is reported that microglia highly adapt to their environment, resulting in diverse temporal and spatial heterogeneous subtypes [42,43]. Concretely, microglia are most heterogenous during postnatal development in a region-dependent manner [44,45].

Pediatric brain tumors exhibit clear correlations with stalled developmental programs and develop in various brain locations [46]. It is speculative if different tumor entities probably adapt to local niches and, thus, progress. For example, microglia subtypes differ in a temporal and region-dependent manner throughout the brain parenchyma. Notably, CD11c+ microglia are highly enriched in the cerebellum during early postnatal development, where they promote the expansion and survival of granule neural precursor cells through insulin-like growth factor 1 (IGF1) secretion [47]. IGF1 was reported to be crucial for tumor growth and migration of (murine) SHH-MB, whose cell of origin is postulated to be cerebellar granule neural precursor cells [48,49].

The impact of tumor-associated microglia/macrophages on tumor progression was reported extensively in various tumor entities [23,41,50].

### 2.2. Brain-Infiltrating Immune Cell Populations

Peripheral blood immune cells, such as natural killer (NK) cells, macrophages (MACs), neutrophils, dendritic cells (DCs), innate lymphoid cells, myeloid-derived suppressor cells (MDSCs) as well as T and B cells can pass into the brain during instances of particularly severe inflammation or trauma [23,24]. In physiological brain development, B cells and T cells reside in the meningeal space and choroid plexus. B cells are involved in oligodendrogenesis and T cells promote the formation of inhibitory synapses by producing IFN-γ [24].

Natural killer (NK) cells, natural killer T cells (NKT), neutrophils and γδ T cells search for pathogens or abnormal cells and then destroy the cell surface using cell toxins. Together with phagocytes, they eliminate pathogens and induce inflammation, which attracts naïve T cells. If this is insufficient to eliminate the pathogen, DCs migrate to the lymph nodes where antigens can be presented to pathogen-specific T-lymphocytes by displaying the antigens on a major histocompatibility complex (MHC). Upon binding to an antigen, CD4+ T cells, CD8+ T cells, and B cells undergo clonal expansion that secrete monoclonal antibodies [51].

In cancer, however, inflammation is not entirely resolved, e.g., due to downregulation of MHC or the lack of neoantigens (as described in the section about immunoediting), and the chronic inflammation leads to recruitment of immunosuppressive immune cells, such as regulatory T cells (Tregs) or myeloid-derived suppressor cells (MDSCs). Further, this immunosuppressed tumor microenvironment impedes T cell infiltration into the tumor core, described as an immunologically “cold” environment [51]. Moreover, peripheral immune cells probably undergo transcriptomic and phenotypic changes upon entering the brain tumor microenvironment [23].

Furthermore, bone marrow-derived monocytes are recruited to the CNS via the chemokine axis CCR2 and CXCR3, where they differentiate into macrophages [42]. It was reported that distinct tumor-associated macrophages (TAM) populations are altered during the course of radiotherapy, response and recurrence [52]. Whether microglia and monocyte-derived macrophages have distinct functions in the brain TME is currently not known, but ontogenically differences were described, e.g., in MB and ATRT [18,53]. In ATRT, TAMs highly interact with tumor cells and support tumor progression by exerting immune-suppressive functions [18]. In contrast, macrophage depletion (using CSF-1 receptor inhibitors) in Gr.3-MB and SHH-MB did not prevent recurrence and metastatic spread [54].

In summary, there is evidence that heterogenous microglia and infiltrating monocyte-derived macrophages are potent regulators of brain tumor development and progression in some brain tumor entities [18,42,52,53]. Despite the growing evidence of macrophages’ pro-tumoral role in tumor biology, the heterogeneity and plasticity of tumor-associated myeloid cells will need to be considered in translational strategies for targeting these innate immune cells. Moreover, since immune cells of the adaptive immune system are encountered in the TME as well, adoptive cellular therapy, like chimeric antigen receptor (CAR)-T cell therapy or vaccine therapy, can be implemented.

## 3. Targeting Immune Cells in Pediatric Brain Tumors

As described in the previous section, tumor-infiltrating innate immune cells play diverse roles in tumorigenesis. Being part of the complex tumor microenvironment (TME), they interact with heterogeneous tumor cell populations, endothelial and stromal cells, and the adaptive immune cells by producing soluble growth factors, cytokines, and chemokines, which support or suppress tumor growth and metastasis [23,50]. While the influence of the TME has been studied very well in adult solid cancers, a comprehensive characterization of the TME in pediatric brain tumors is still lacking. In the following section, we give an explorative overview of immunophenotyping studies in pediatric brain tumors.

### 3.1. Immunophenotyping Studies of the Tumor Microenvironment in Pediatric Brain Tumors

In recent years, the number of immunophenotyping studies in pediatric brain cancers consistently increased. A literature search in the bibliographic database of PubMed was performed for the period from 2000 to 2021 using the following keywords: (“pediatric brain tumors” or “embryonal brain tumors”) and (“immune” or “microenvironment”). The titles and abstracts of the papers were evaluated concerning microenvironment-related or immunophenotyping studies in pediatric brain tumors.

Most studies relied on immunohistochemical (IHC) analysis of conserved patient tissue or multicolor fluorescence-activated cell sorting (FACS) analysis of fresh material. Since IHC and FACS analyses are based on limited cell phenotype markers, and tissue is disaggregated for flow cytometry, methods for deconvolution of immune cell signatures from bulk RNA expression data have become more prominent over the years. Algorithm like ESTIMATE, CIBERSORT or Microenvironment Cell Populations (MCP)-counter quantify immune and stromal cell populations based on defined marker gene expression signatures [55,56,57]. Further progress on deconvolution approaches was made on genome-wide DNA methylation data, introducing MeTIL, MethylCIBERSORT and DIMEimmune [58,59,60].

Grabovska and colleagues [21] used MethylCIBERSORT to recapitulate the tumor microenvironment of over 6000 primarily pediatric CNS tumors by interrogating 12 broad cell types, namely B cells, CD4+ T cells, CD8+ T cells, CD4+/FOXP3+ Treg, NK cells, monocytes, neutrophils, eosinophils, endothelial cells, glial cells, neurons and cancer cells [21]. The total amount of infiltrating cells was significantly higher in low-grade gliomas (LGGs) than in high-grade tumors such as embryonal tumors (MB, ATRT, ETMR). In addition, key molecular features of a tumor type, such as MYC amplification in Gr.3-MB or H3.3G34 mutations in HGG, were significantly associated with high numbers of CD8+ T cells and B cells, and CD8+ T cells, respectively. Thus, these mutations potentially have an impact on the immunological antitumor response. Furthermore, they described three distinct TME classes, which strongly correlate to tumor subgroups and WHO grades. In detail, 86% of grade IV tumors were classified to panCNS_IC2_ (CD8+ T cells high; CD4+ T and NK cells low) and 87% of grade I tumors belonged to panCNS_IC3_ (monocytes high; CD8+ T cells low). PanCNS_IC1_ (Tregs high; CD8+ T cells low) displayed a balanced grade I–IV tumor distribution. Lastly, significant prognostic relationships were found in infant SHH-MB (B cell amount significantly greater), low-risk Gr.3-MB (Treg significantly associated with poor survival) and pediatric HGG (monocytes significantly enriched in tumors with MAPK mutations) [21]. Safaei et al. [60] developed DIMEimmune for differential methylation analysis for immune cell estimation [60]. This study calculated estimations of CD4+, CD8+ and tumor-infiltrating lymphocytes (TILs) for glioma, MB, ATRT and EPN methylation data. Concordant with the previous study, pHGG showed higher scores of TILs than pLGG and IDH-mutated gliomas. ATRT were highly infiltrated by lymphocytes, most prominently in the MYC subgroup, while there were overall only a few TILs in MB subgroups. Among EPN, PFA-EPN had the largest estimated number of TILs [60].

Recently, a large immune profiling study from transcriptomic data of 495 pediatric gliomas (PGs) was performed [61]. Basically, PGs were classified into three immune subtypes, namely immune hot (IS-I), immune altered (IS-II), and immune cold (IS-III). IS-I tumors (more pLGGs, no DIPGs), characterized by substantial immune infiltration and high expression of immune checkpoint molecule (ICM), had a favorable prognosis, whereas IS-III tumors (large fractions of pHGGs and DIPGs) showed weak immune infiltration and low ICM expression, had a dismal prognosis and poor immunotherapy responsiveness. The IS-II classification represented a transitional stage. Immune classification was also correlated with somatic mutations, copy number alterations, and molecular pathways related to tumorigenesis, metabolism, and immune responses [61]. The finding that LGGs are characterized by greater immune cell infiltration, especially T cells, compared to HGGs are in line with other publications [17]. However, even among LGG, T cell infiltration was highly variable and subgroup-dependent, with greater T cell density in pleomorphic xanthoastrocytoma (PXA) and ganglioglioma (GG) [17]. A gene-expression study comparing adult and pediatric HGG found that age did not shape the tumor microenvironment but mutational and transcriptional phenotypes [62].

Another comprehensive study intercorporate proteomics inclusive of the genomics, and transcriptomics of a cohort of 218 tumor samples representing LGG, EPN, HGG, ATRT, MB, GG and craniopharyngioma (CP). This study reveals downstream effects of genetic alterations not evident in transcriptomics. ATRT, MB and EPN showed lower immune infiltration, while LGG had higher immune infiltration [63].

These data demonstrate, that in silico deconvolution methods are feasible to be implemented into a diagnostic assessment of patients for personalized therapies since methylation data are increasingly used for molecular diagnostics. Nonetheless using these methods, one potentially misses tumor-specific characteristics of infiltrating cells. More depth into TME analyses can be achieved by single-cell transcriptomics (scRNA-seq). Marker genes for distinct immune cells can be easily applied to identify these different cell types, while having additional information on tumor-specific signatures of these cells. Until now, only few single-cell studies were obtained from pediatric brain tumors [15,18,49,53,64].

ScRNA-seq analysis of murine ATRTs revealed elevated immune cell infiltration in ATRT-MYC and ATRT-SHH, predominantly composed of myeloid cells and T cells [15,18]. Further, these studies unraveled a heterogeneity of myeloid cells, having a critical role on tumor progression and chemoresistance [15,18]. These myeloid cells impact T cells, which recapitulated many of the T cell subpopulations identified in several immunogenic adult cancer types, like lung adenocarcinoma and melanoma [15]. In addition, CD8+ T cells were clonally expanded, revealing immunogenicity of rhabdoid tumors [15].

Furthermore, several studies examined the influence of infiltrating immune cells on patients’ prognosis [18,19,20,22,65] (Table 1). For example, among MB subgroups TAMs are enriched in SHH-MB patients, having an antitumoral role [66]. In contrast, CD68+ expression was significantly associated with a worse prognosis in ATRT patients, where ATRT-SHH and ATRT-MYC were highly infiltrated by CD68+ cells [18]. Finally, a selection of immune profiling studies in pediatric brain tumors is listed in Table 2.

**Table 1:** Associations between immune microenvironment and molecular features/prognosis in pediatric brain tumors.

Entity	Immunological Profile/Immune Population	Associated Molecular Features	Prognosis	Sample Cohort	Study
pLGG, pHGG	Hot (IS-I): more pLGGs, no DIPGs.	BRAF mutation (69.6%)	MS ^1^: 29.8y/>18y	384 from CBTTC/111 from ICGC	[61]
	Altered (IS-II): transitional stage.	SVIL mutation (55.5%)	MS ^1^: 19.2y/13.3y		
	Cold (IS-III): large fractions of pHGGs, DIPGs.	CACNA1A mutation (74.2%)	MS ^1^: 14.5y/1.99y		
	Monocytic lineage expression ↑		Improved OS ^2^	113	[62]
pHGG	Monocytes ↑	MAPK mutation		143	[21]
	NK cells ↑	G34/WT-C	Poor OS ^2^: 3.0		
	B cells ↓	WT-A	Poor OS ^2^: 4.3		
	CD8 T cells ↑	hypermutator tumors (MMRD ^4^; POLE/POLD1 mutations); BRAF^V600E^ or NF1		113	[67]
Gr.4-MB	Monocytes ↑			408	[21]
SHH-MB ^3^	Tregs ↑		Poor OS ^2^: HR 1.7		
Gr.3-MB	CD8 T cells ↑; B cells ↑; Tregs ↓	MYC amplification	Poor OS ^2^: HR 3.3		
SHH-MB	AIF1 expression (MAC/MG ^5^) ↑		Improved OS ^2^	172	[66]
	B cells ↑		Improved OS ^2^	35	[21]
ATRT	CD68+ MAC/MG ^5^ ↑		Poor OS ^2^: HR 11.9	34	[18]
	CD4/8 T cells ↑	PBRM1 ↑	Improved OS ^2^	33	[68]
	CD163+ macrophages ↑	PBRM1 ↑	Poor OS ^2^		

^1^ Median survival; ^2^ overall survival; ^3^ infant; ^4^ mismatch repair deficiency; ^5^ macrophages/microglia. ↑: High cell number/expression, ↓: Low cell number/expression

Outlining the results of all mentioned immunophenotyping studies for a consensus output is quite challenging because of the different methods, sample cohorts, and the choice of read-out. In addition, each method has its limitations, and some study results are conflicting. Larger cohorts of well-defined molecular subtypes are needed to assess the prognostic role of immune cells in pediatric brain tumors.

Notwithstanding, it became apparent that the tumor microenvironment is a promising perspective to overcome therapy resistance. In the next chapter, we further consider essential aspects for implementing immunotherapeutic targets to conventional therapy. First, we discuss the impact of tumor-specific alterations in epigenetic modifiers and signaling pathways on the tumor immune microenvironment. Second, we summarize how standard therapeutic approaches (e.g., chemotherapy and radiotherapy) influence tumor-associated immune cells, and third, we give insights into therapeutic strategies targeting tumor-associated immune cells to implement innovative treatment strategies for children with brain cancers.

### 3.2. Impact of Epigenetic Dysregulation and Aberrant Signaling Pathways in Tumors on Immune Cells

Some pediatric brain tumors are characterized by alterations in epigenetic modulators, e.g., mutations in chromatin remodeling complexes like SWItch/Sucrose Non-Fermentable (SWI/SNF) [80]. Deregulation of epigenetic mechanisms results in aberrant gene expression, contributing to tumor formation and presumably altered immune response [81]. A genome-scale CRISPR-Cas9 screening of melanoma cells identified PBRM1, ARID2, and BRD7 of the chromatin-remodeler PBAF (polybromo-associated BAF) to contribute to the tumor cell resistance by blocking the effects of cytotoxic T cells [82]. Alterations in the chromatin-remodeling complexes of the SWI/SNF family (BAF, PBAF), particularly in the subunits SMARCB1 (INI1) and SMARCA4, are the primary driver event for rhabdoid tumor development [83,84]. Recently, a study elucidating the SWI/SNF complex heterogeneity in ATRT and extracranial rhabdoid tumors (eRT) found a correlation between the PBAF subunit gene expression and immune cell infiltration in ATRT and eRT. *PBRM1*, which encodes a component of the PBAF complex, regulates the expression of immune-related genes, like interleukins (e.g., IL-13 and IL-16) and tumor necrosis factor alpha, in rhabdoid tumors. The level of *PBRM1* expression was inversely correlated with the degree of CD8+ cytotoxic T cell infiltration. PBRM1-low patients (10% ATRT-SHH, 40% ARTR-MYC, 50% ATRT-TYR) had higher T cell infiltration and a better outcome than PRBM1-high patients (around 60% ATRT-SHH, 20% ARTR-MYC, 20% ATRT-TYR). Conversely, CD163+ macrophages were significantly increased in PBRM1-high patients. The authors suggest that immune checkpoint inhibition can be a potential therapeutic strategy in PBRM1-low rhabdoid tumors [68]. Another hint for a potential connection between epigenetic dysregulation and TME is found in pediatric glioma. Histone mutant (H3 K27M, H3 G34RV) tumors were considerably immune “cold” as defined by a lack of TILs, especially CD8+ T cells, and had a poor outcome in comparison to non-histone mutated HGG [67,85].

Besides epigenetic dysregulation, various signaling pathways are activated by genetic events in pediatric brain tumors, e.g., MAPK in LGG, SHH and WNT signaling in MB and ETMR, SHH and TYR in ATRT [3,7,86]. Subgroup-specific immune cell infiltration in ATRT, MB and glial tumors implies that specific molecular aberrations probably impact on the immune microenvironment [17,18,76].

Generally, there is an ongoing debate if oncogenic molecular aberrations can be sufficient to drive immune exclusion in tumors. For example, tumors with active WNT/β-catenin signaling are characterized by lower levels of T cell infiltration. Thus, targeting this pathway would antagonize not only the aberrant signaling pathway but also restore T cell infiltration [87]. Though, in MB subgroups, lymphocyte infiltration was low in all four MB subgroups, not exclusively in WNT-MB [71,77]. In a proteomics-based study of pediatric brain tumors, a non-inflammatory microenvironment exhibited upregulation of WNT/β-catenin signaling, regulation of apoptosis and proteasome. This “cold” phenotype was attributed to MB samples without subgroup specification [63]. Still, there are some moderate differences within MB subgroups according to their TME profile [76]. A significantly higher proportion of TILs, cytotoxic T cells, and B cells and a lower infiltration of Tregs were associated with MYC amplification in Gr.3-MB [21].

Further, the immunomodulatory role of SHH/GLI signaling was described in various inflammatory and malignant diseases. Elevated SHH signaling induces immunosuppressive mechanisms such as recruitment of MDSCs and MACs [88,89]. Moreover, WNT and SHH pathways are associated with a stemness-enriched tumor profile, and stemness, in turn, was linked to resistance to immune-mediated destruction [89]. ATRT-SHH tumors showed a higher stemness score than other ATRT subtypes and were highly infiltrated by myeloid cells [18,19]. In glial tumors, CD3+ T cell infiltration correlates inversely with the expression of *SOX2*, an embryonal stem cell marker commonly expressed by glial tumors [17].

Another association between molecular aberration and infiltrating cell estimation was found in gliomas. LGG BRAF^WT^-rich, LGG BRAF^Fusion^-rich, and CP/LGG BRAF^V600E^ had higher immune infiltration [63]. Further, M1 macrophages and M2 microglia were upregulated in BRAF^Fusion^ compared with the wildtype. BRAF^Fusion^ promoted more M2 microglia, whereas BRAF^V600E^ promoted more M2 macrophages [79]. Besides PXA-like tumors, which harbored either BRAF- or NF1-driven MAP-kinase alterations, hypermutated cases had the highest amount of CD8+ T cells [17,67]. Further, PD-L1 expression was independent of BRAF^V600E^ mutational status [79].

### 3.3. Immunomodulation by Chemotherapy, Radiotherapy and Targeted Anticancer Agents

As outlined before, significant advances have been made in the field of molecular biology in pediatric neuro-oncology. These studies generated valuable molecular sub-classification of pediatric brain tumor entities, established new guidelines for patient risk stratification and identified some candidate target genes (reviewed in [5,8]). Nevertheless, we have little knowledge about the function of infiltrating immune cells in pediatric brain tumors and their potential modulation by radiation, chemotherapy, and targeted molecular therapies.

Ionizing radiation induces DNA damage in quickly dividing tumor cells, sparing non-transformed stromal cells. Observations of lesions outside the radiation area revealed that systemic antitumor effects take place at the lesion borders. These effects include the induction of immunogenic cell death (ICD), characterized by exposed danger-associated molecular patterns (DAMP) that recruit immune cells, and increased MHC I expression. This phenomenon could be a strategic approach for converting immunologically “cold” tumor environments into “hot” environments [90]. Additionally, chemotherapeutic agents can promote the initiation of ICD of tumors by enhancing the antigenicity of tumor cells. This chemotherapy-induced ICD is highly dependent on the agent and the specific composition of the TME [91]. For example, chemotherapy treatment of glioblastoma with widely used temozolomide (TMZ) has limited efficacy as a monotherapy. Still, it has several immunomodulatory effects, like enhancing antigen-specific T cell proliferation [92].

Besides that, there are hints that radiation and chemotherapy provoke the infiltration of suppressive monocyte-derived macrophages (MDM), which have a role in acquired resistance after therapeutic intervention [18,52,53]. In glioma, MDM and microglia were altered substantially in number and phenotype during radiation therapy, response after irradiation, and recurrence. By combining radiotherapy with colony-stimulating factor–1 receptor (CSF1R) inhibition for TAM depletion, overall survival was enhanced in preclinical glioma models [52]. In a relapse xenograft model of ATRT-MYC, Melcher et al. [18] showed that MDM contribute to chemotherapy resistance and tumor relapse. In murine SHH-MB radiation therapy, but not targeted therapy (SHH-pathway inhibitor GDC-0449), recruited immunosuppressive MDM that reduced T cells and neutrophils numbers [53].

Furthermore, it should also be considered that some anticancer agents used in early clinical studies for the treatment of pediatric brain tumor patients, e.g., for epigenetic reprogramming, have immunomodulating properties that can hamper the therapeutic efficacy of targeted therapies. The stimulatory or suppressive effects on immunity can originate from the effect of the drug on tumor cells, as well as from the drug’s ability to modulate immune cells directly [93].

For example, EZH2 methyltransferase activity inhibitor GSK126 supported the expansion of immunosuppressive MDSCs in two carcinoma mouse models. This epigenetic intervention restrained tumor growth in immunocompromised mice but not in immunocompetent mice [94]. Indeed, regression of INI-deficient rhabdoid tumors after EZH2 inhibition was observed based on preclinical evaluation with human cancer cell lines and xenograft models in immunodeficient mice [95,96]. A Phase 1 study of the EZH2 inhibitor tazemetostat in pediatric patients with relapsed or refractory INI1-negative tumors (NCT02601937) is ongoing, though interim results presented an overall response rate of 17% [97]. In a phase 2 study of adult patients with tazemetostat (NCT02601950), a stable disease as the best overall response was observed in 7 of 31 patients in cohort 1 (rhabdoid tumors) [98]. In the same study, 15% of 62 epithelioid sarcoma patients (cohort 5) had an objective response [99]. It remains speculative if EZH2 inhibition restrains antitumoral immune response, and if a multimodal treatment is decisive to maximize the efficacy of targeted anticancer agents. For example, 5-fluorouracil- or gemcitabine-based chemotherapy that deplete MDSCs and/or limit their immunosuppressive functions stand out as combinatorial partners for EZH2 inhibitors [91]. In DIPG (H3K27M mutation), the use of EZH2 resulted in decreased cancer cell invasion, increased microglial phagocytosis, and tumor cell death, but not tumor cells per se contribute to the observed tumor repression after EZH2 inhibition, but rather the induced transition of microglia to an antitumoral phenotype [100].

Epigenetic reprogramming with histone deacetylases (HDAC) inhibitors upregulate PD-L1 and PD-L2 in preclinical melanoma models. Treated mice with a combinatorial PD-1 blockade and HDAC inhibitor showed decreased tumor progression and better survival than control mice and single-agent treated mice [101]. In addition, this treatment combination leads to general changes in the TME, such as enhanced immune cell infiltration, elevated T cell memory, and a significant reduction of M2 macrophages [102].

The spectrum of immunomodulation by cyclin-dependent kinases (CDKs) inhibitors like CDK4/6 is broad. Various inhibitors mediate immunostimulatory effects (secretion of pro-inflammatory cytokines; enhanced antigenicity of malignant cells; upregulation of PD-L1) and act directly on immune cells, e.g., depleting Tregs [93].

Considering the adverse effects of chemo- and radio-therapy and the immunomodulatory effects of targeted therapies, a synergistic treatment regimen involving immunotherapy could be promising to improve the efficacy of standard and genome-based molecular therapeutics.

### 3.4. Immunotherapeutic Strategies

Immunotherapy can be broadly categorized in monoclonal antibody (mAb) therapy and adoptive cellular therapy, which include immune checkpoint inhibition (ICI), chimeric antigen receptor (CAR)-T cell therapy, vaccine therapy, and oncolytic virus therapy. Present therapeutics targeting TME have focused predominantly on T cells, but the majority of patients do not respond to ICI or T cell therapy as a result of elevated immunosuppressed microenvironment or inadequate antigenic load within the tumor [103]. Due to extensive research on the TME, it is now evident that the innate immune cells indirectly influence the TME by controlling T cell fate. These innate immune cell types, including macrophages, DCs, neutrophils, MDSCs, NK, and ILCs, critically sculpt the TME [23].

#### 3.4.1. Immune Checkpoint Inhibition (ICI)

**Figure 1:**
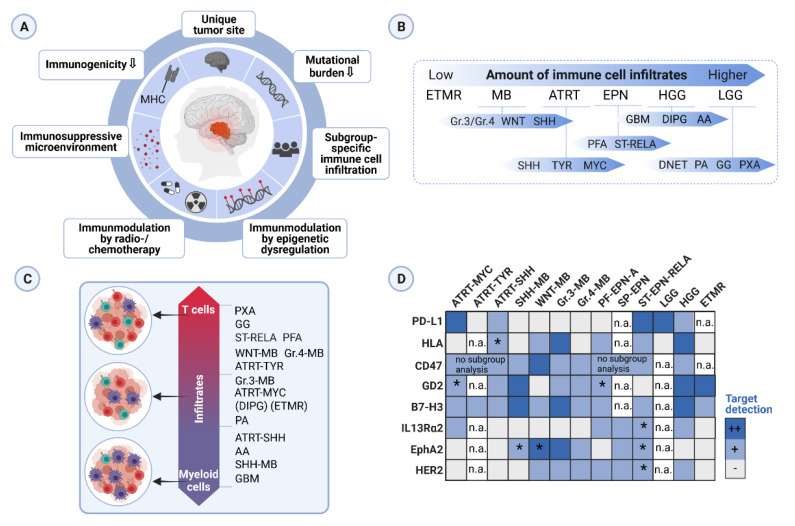
Graphical summary of the key topics in this review. (**A**) Hallmarks of immunogenicity of pediatric brain cancer. (**B**) Ranking of pediatric brain tumor entities according to the amount of immune cell infiltration. Subgroup ranking is related to the respective entity. Abbreviations: ETMR (embryonal tumors with multilayered rosettes), MB (medulloblastoma), ATRT (atypical teratoid rhabdoid tumor), EPN (ependymoma), HGG (high-grade glioma), LGG (low-grade glioma), GBM (glioblastoma), DIPG (diffuse intrinsic pontine glioma), AA (anaplastic astrocytoma), PFA (posterior fossa, pediatric-type), ST-RELA (supratentorial, *RELA* fusion), DNET (dysembryoplastic neuroepithelial tumor), PA (pilocytic astrocytoma), GG (ganglioglioma), PXA (pleomorphic xanthoastrocytoma). (**C**) Ranking pediatric brain tumor entities along a scale of two broadly defined immune cell infiltrate categories, namely myeloid-enriched (violet) or T cell-enriched (red) infiltration. (**D**) Illustration of a selection of immunotherapeutic targets evaluated in several pediatric brain tumor studies. The color code represents a qualitative evaluation. Abbreviations: PF-EPN (posterior fossa ependymoma), SP-EPN (spinal ependymoma), n. a. (not analyzed in depicted studies), * (contradicting results due to different methods used in the depicted studies). Findings in (**B**–**D**) were retrieved from [15,16,17,18,21,61,63,78,106,108,109,111] descriptively. Created with BioRender.com.

In solid adult malignancies, tumor mutational burden (TMB; number of DNA mutations per megabase in a tumor genome) and the presence of TILs are considered as biomarkers for the efficacy of ICI [11]. Immune checkpoint molecules like programmed cell death protein 1 (PD-1), indoleamine 2,3-dioxygenase (IDO), or cytotoxic T lymphocyte-associated antigen 4 (CTLA-4) are regulators of the adaptive immune response, which prevent exaggerated T cell activity when bound to their receptors on the T cell surface [104]. Thus, tumor cells that express, e.g., the ligand of PD-1, PD-L1, can inhibit T cell response. Compared to adult cancer or the adult counterpart, pediatric tumors have a low TMB [14]. A recent comprehensive study examining TMB and driver mutations in 723 pediatric brain tumors (covered inter alia LGG, HGG, MB and embryonal tumors, EPN) confirmed the generally low TMB in pediatric brain cancers (91.8% of the tumors). Tumors with a high TMB (2.1%) were high-grade gliomas and had alterations in *TP53* and concurrent mismatch repair gene alteration [13]. Hence, ICI may only have significant application for a small proportion of pediatric brain tumor patients. Moreover, PD-L1 expression is low frequented and heterogenous in pediatric brain tumors (Table 2) (Figure 1) [22,75,79,105].

#### 3.4.2. Targets for CAR-T Cell Therapy and Innate Immune Cells

A strong antitumor T cell response requires an immunogenic antigen not recognized as a “self” molecule. Tumor neoantigens derived from genetic alterations are potential T cell targets, but the generally low mutational load in pediatric tumors impedes the number of targets for immunotherapy with T cells expressing tumor-specific chimeric antigen receptors (CARs) [13]. Still, CAR-T cell therapy seems to be more promising than ICI since MB, ATRT, EPN and ETMR showed low expression of MHC-class I molecules, which are indispensable for HLA-based immune response by conventional αβTCR T cells [106]. A broad characterization of the five potential CAR-T cell targets IL13Rα2, HER2, EPHA2, B7-H3 and GD2, investigated in 49 patient-derived orthotopic xenografts of pediatric brain tumors, was performed by Haydar et al. [106] (Table 3). The most promising target was B7-H3, having the highest expression in MB and HGG, followed by GD2. Another screen of B7-H3 expression in solid pediatric tumors and brain neoplasms showed similar results [107]. However, Haydar et al. did not detect B7-H3 in ATRT by immunohistochemical examination, which stands in contradiction to the study of Theruvath et al. [108]. Aside from that, locoregional administration of B7-H3-CAR-T cells mediated potent antitumor response against cerebral ATRT and MB xenografts, and local or systemic administration induced tumor regression in patient-derived xenografts and immunocompetent murine glioma models resulting in a significant survival advantage [106,107,108]. In primary, metastatic, and recurrent Gr.3-MB and PFA-EPN xenografts, intrathecal delivery of EPHA2-, HER2- and IL13Rα2-CAR-T cells were validated as an effective treatment. Furthermore, administration in the cerebrospinal fluid, alone or combined with azacytidine, was highly effective for multiple metastatic mouse models of Gr.3-MB and PFA-EPN [109].

Another therapeutic target, which is probably more relevant for pediatric brain tumors with elevated myeloid cell infiltration, is cluster of differentiation 47 (CD47) on tumor cells that binds to signal-regulatory protein alpha (SIRPα) on myeloid cells and inhibits macrophage-induced phagocytosis [110]. Blocking this pathway with anti-CD47 antibody inhibited tumor growth in patient-derived xenografts of Gr.3-MB, ATRT, PNET, GBM and diffuse midline glioma (DMG) [111] (Table 3).

**Table 3:** Targets for immunotherapy in pediatric brain tumors.

Entity	Targets	Therapy Approach	Study
GBM, MB, EPN	EGFRvIII	CAR T cells	[112]
MB, EPN	IL13Rα2	CAR T cells	[109,113]
MB, EPN	HER2	CAR T cells	[109,114,115]
MB, EPN	EPHA2	CAR T cells	[109]
SHH-MB	CD1d	Vα24-invariant (type-I) NKT cells	[116]
MB	PRAME	CAR T cells (SLL TCR T cells with inducible caspase-9 gene)	[117]
MB	No target	allogeneic cord blood-derived NK cells expressing a dominant negative TGF-β receptor	[118]
SHH-MB	CSF1R	CSF1R inhibitor (PLX5622) for TAM depletion	[119]
ATRT, MB, ETMR, EPN, HGG	B7-H3	CAR T cells	[106,107,108]
ATRT, MB, ETMR, EPN, HGG	GD2	CAR T cells	[106,120]
Gr.3-MB, ATRT, PNET, GBM, DMG	CD47	α-CD47 against tumor cells	[111]
ATRT	PD-1	α-PD-1 against PD-1+ immune cells	[15]

As mentioned before, dendritic cells (DCs) link innate and adaptive immunity by presenting antigens to T cells and trigger T cell response [51]. Thus, DCs present a promising target for immunotherapy. In ATRT, a small cohort of seven patients received autologous DCs loaded with tumor-lysate. Three patients survived long-term, and tumor-specific CD8+ T cell responses were reported [121]. Another study used tumor RNA-loaded DCs to treat seven pediatric brain tumor patients, of which three clinically responded, but no lymphocyte-mediated antitumor response was detected [122]. Further, in a study of 45 children with different brain tumors, patients were vaccinated with tumor-lysate-loaded DCs. The DC vaccine therapy was especially beneficial for ATRT and HGG [123].

Moreover, DCs can be loaded with peptides and proteins, as well. Recently, a comprehensive mass spectrometry study of MHC-class I and -class II peptides on 23 ATRTs reported possible targets for T cell recognition [70]. Analysis of the ATRT cell surface, which includes tumor-associated antigens derived from canonical non-mutated or overexpressed natural proteins and non-canonical or cryptic sequences (from noncoding regions), revealed 55 HLA-class I, 139 HLA-class II tumor-specific peptide sequences, and 61 HLA-class I peptides derived from non-canonically translated peptides. Comparative analyses to other tumor entities showed overlap with the cryptic ligandome of glioblastomas, whereas no similarity was found with extracranial tumors. Testing the immunogenicity of the peptides, over 80% of ATRT-specific peptides were able to prime CD8+ T cells. Moreover, over 50% of these peptides were also recognized by glioblastoma-derived T cells but not healthy T cells. The authors conclude that the results of ATRT’s immunopeptidome could be paradigmatic for other low mutated pediatric cancers and suggested including cryptic peptides into therapeutic vaccines [70].

Lastly, oncolytic virus therapy is another immunotherapeutic approach by which viruses target and kill cancer cells selectively while leaving normal tissues unaffected. For example, the oncolytic adenovirus Delta-24-RGD induced dose-dependent tumor cytotoxicity and increased CD8+ T cell infiltration in ATRT and other embryonal brain tumor models, resulting in significantly increased survival [124].

Hence, although promising targets for mAb and CAR-T cell therapy have been found in preclinical studies (Table 3) and vaccine therapies showed promising outcomes, further investigations in clinical trials for pediatric brain tumors are still scarce. Safety risks and adverse effects, such as cytokine releasing syndrome as the most common adverse event following CAR-T therapy, require close monitoring and management strategies to be set up [125].

### 3.5. Challenges in Clinical Translation

Many advancements have been made in developing immunotherapeutic approaches for brain tumors, especially in adult cancers. As presented in this review, pediatric brain tumors are not as “cold” as assumed. Still, it is just the beginning of a deeper evaluation and consideration of the pediatric brain tumor microenvironment for implementation into stratifications and therapies. More research needs to be done to understand the TME, tumor epitopes, the heterogeneity among entities and subgroups, as well as different BBB phenotypes. Further, there is a lack of appropriate model systems.

Several challenges need to be addressed: (1) low mutational load of pediatric brain tumors and potentially limited immunogenicity, (2) differences in adult and pediatric immune infiltration, and differences even throughout childhood development [29], (3) combinatorial treatment regimens and interactions, and iv. management of immunotherapy toxicity since the CNS is particularly vulnerable to structural and functional damage in response to systemic inflammation.

## 4. Conclusions

There is no doubt that therapies reducing the side effects of highly intensive chemo- and radio-therapy are needed in the treatment regimen of pediatric brain tumor patients. The potential of immunotherapy in solid pediatric cancers is yet not fully uncovered. However, recent research efforts shed light on the immune-biological processes and laid the ground for in-depth analysis of the tumor microenvironment of all pediatric brain tumors. Besides analyzing the specific molecular and genetic characteristics of each patient’s tumor, immune correlate studies should be considered prior to exposure to chemotherapy to maximize treatment efficacy of combined treatment regimen with immunotherapeutic approaches.

## Figures and Tables

**Table 2:** Immune profiling studies in pediatric brain tumors.

Entity	Findings	Sample Origin	Techniques
ATRT	▪ Immune cell infiltration with CD68+ microglia/macrophages and CD4+/CD8+ T cells [69]	H	IHC
	▪ Immune cell infiltration with myeloid and T cells; ATRT-MYC highly infiltrated; clonally expanded T cells [15]	H, M	IHC, FACS, RNAseq, sc-RNAseq
	▪ Subgroup-specific immune cell infiltration; CD68+ cells as negative prognostic factor [18]	H, M	IHC, scRNA-seq
	▪ CD8+ T cell infiltration higher in ATRT-MYC than ATRT-TYR/-SHH; PD-L1 and PD-1 expression [16]	H	RNAseq, IHC
	▪ Analysis of naturally & cryptic presented HLA-class-I and class-II ligands [70]	H	MS
MB	▪ Immunosuppressed myeloid cells; T cells are less frequent than in PA and EPN [71]	H	FACS, RNAseq
	▪ CD163 expression is most enriched in SHH-MB compared to other MB subtypes; CD1d expression in a subset of infantile MB [72]	H	RNAseq, IHC
	▪ Increased expression of inflammation-related genes; greatest number of CD163+ TAMs in SHH-MB across MB subtypes [73]	H	RNAseq, IHC
	▪ CD4+, CD8+ T cells, MDSCs, DCs and TAMs in SHH-MB > Gr.3-MB; CD8+ PD-1+ T cells in Gr.3-MB > SHH-MB; Gr.3-MB respond to PD-1 blockade, MB-SHH not [74]	M	In vivo, FACS, IHC
	▪ No PD-L1 expression in four MBs [75]	H	IHC
	▪ No PD-L1 expression in 26 MBs; no correlation of TILs with overall survival; expression of granzyme inhibitor SERPINB1 was associated with better survival [22]	H	IHC
	▪ Subgroup-specific immune microenvironment [76]	H	RNAseq
	▪ Antitumoral role of TAMs in SHH-MB [66]	H, M	RNAseq, in/ex vivo, FACS
	▪ Increased expression of immune-related genes in SHH-MB compared to other MB subtypes; CD8+ T cells and neutrophils enriched in G4-MB; no PD-L1 expression in 19 MBs [77]	H	RNAseq, IHC
	▪ TAMs in SHH-MB are of microglial origin and monocyte-derived; radiation therapy, but not targeted therapy, recruited immunosuppressive monocyte-derived macrophages that reduced T cells and neutrophils [53]	H, M	RNAseq, scRNAseq, in vivo, IHC, RNA in situ hybridization, FACS
EPN	▪ Higher numbers of T cells and myeloid cells compared to MB and GBM [71]	H	FACS, RNAseq
	▪ High expression of PD-L1 in ST-EPN-RELA with PD-1+ CD4+ and CD8+ T cells [78]	H	RNAseq, WB, IHC, FACS
LGG	▪ T cell infiltrates higher in LGG compared to HGG; Within LGG greater T-cell density in PXA and GG; CD3+ T cell infiltration correlates inversely with SOX2 expression [17]	H	Multiplex IHC, Single-cell mass cytometry
	▪ PD-L1 expression independent of BRAF^V600E^ mutational status [79]	H	IHC, in vitro
	▪ Highest CD8+ T-cell density in PXA and hypermutator LGGs. Histon mutant tumors immune “cold” [67]	H	RNAseq, IHC
HGG	▪ T cells and myeloid cells are less frequent than in PA and EPN [71]	H	FACS, RNAseq
	▪ Immunologic profiling of pediatric and adult HGGs [62]	H	RNAseq
	▪ Immunologic profiling of pediatric LGG and HGG [61]	H	RNAseq

## References

[1] Johnson K.J., Cullen J., Barnholtz-Sloan J.S., Ostrom Q.T., Langer C.E., Turner M.C., McKean-Cowdin R., Fisher J.L., Lupo P.J., Partap S. (2014). Childhood Brain Tumor Epidemiology: A Brain Tumor Epidemiology Consortium Review. Cancer Epidemiol. Biomark. Prev..

[2] Louis D.N., Perry A., Reifenberger G., von Deimling A., Figarella-Branger D., Cavenee W.K., Ohgaki H., Wiestler O.D., Kleihues P., Ellison D.W. (2016). The 2016 World Health Organization Classification of Tumors of the Central Nervous System: A summary. Acta Neuropathol..

[3] Northcott P.A., Korshunov A., Witt H., Hielscher T., Eberhart C.G., Mack S., Bouffet E., Clifford S.C., Hawkins C.E., French P. (2010). Medulloblastoma Comprises Four Distinct Molecular Variants. J. Clin. Oncol..

[4] Zhang J., Wu G., Miller C.P., Tatevossian R.G., Dalton J.D., Tang B., Orisme W., Punchihewa C., Parker M., Qaddoumi I. (2013). Whole-genome sequencing identifies genetic alterations in pediatric low-grade gliomas. Nat. Genet..

[5] Cacciotti C., Fleming A., Ramaswamy V. (2020). Advances in the molecular classification of pediatric brain tumors: A guide to the galaxy. J. Pathol..

[6] Capper D., Jones D.T.W., Sill M., Hovestadt V., Schrimpf D., Sturm D., Koelsche C., Sahm F., Chavez L., Reuss D.E. (2018). DNA methylation-based classification of central nervous system tumours. Nature.

[7] Gröbner S.N., Worst B.C., Weischenfeldt J., Buchhalter I., Kleinheinz K., Rudneva V.A., Johann P.D., Balasubramanian G.P., Segura-Wang M., Brabetz S. (2018). The landscape of genomic alterations across childhood cancers. Nature.

[8] Fangusaro J., Bandopadhayay P. (2021). Advances in the classification and treatment of pediatric brain tumors. Curr. Opin. Pediatr..

[9] Rutkowski S., Bode U., Deinlein F., Ottensmeier H., Warmuth-Metz M., Soerensen N., Graf N., Emser A., Pietsch T., Wolff J.E.A. (2005). Treatment of Early Childhood Medulloblastoma by Postoperative Chemotherapy Alone. N. Engl. J. Med..

[10] Vinchon M., Baroncini M., Leblond P., Delestret I. (2011). Morbidity and tumor-related mortality among adult survivors of pediatric brain tumors: A review. Child’s Nerv. Syst..

[11] Lieberman N.A.P., Vitanza N.A., Crane C.A. (2018). Immunotherapy for brain tumors: Understanding early successes and limitations. Expert Rev. Neurother..

[12] Paugh B.S., Qu C., Jones C., Liu Z., Adamowicz-Brice M., Zhang J., Bax D.A., Coyle B., Barrow J., Hargrave D. (2010). Integrated molecular genetic profiling of pediatric high-grade gliomas reveals key differences with the adult disease. J. Clin. Oncol..

[13] Patel R.R., Ramkissoon S.H., Ross J., Weintraub L. (2020). Tumor mutational burden and driver mutations: Characterizing the genomic landscape of pediatric brain tumors. Pediatr. Blood Cancer.

[14] Vogelstein B., Papadopoulos N., Velculescu V.E., Zhou S., Diaz L.A., Kinzler K.W. (2013). Cancer Genome Landscapes. Science.

[15] Leruste A., Tosello J., Ramos R.N., Tauziède-Espariat A., Brohard S., Han Z.Y., Beccaria K., Andrianteranagna M., Caudana P., Nikolic J. (2019). Clonally Expanded T Cells Reveal Immunogenicity of Rhabdoid Tumors. Cancer Cell.

[16] Chun H.J.E., Johann P.D., Milne K., Zapatka M., Buellesbach A., Ishaque N., Iskar M., Erkek S., Wei L., Tessier-Cloutier B. (2019). Identification and Analyses of Extra-Cranial and Cranial Rhabdoid Tumor Molecular Subgroups Reveal Tumors with Cytotoxic T Cell Infiltration. Cell Rep..

[17] Robinson M.H., Vasquez J., Kaushal A., MacDonald T.J., Velázquez Vega J.E., Schniederjan M., Dhodapkar K. (2020). Subtype and grade-dependent spatial heterogeneity of T-cell infiltration in pediatric glioma. J. Immunother. Cancer.

[18] Melcher V., Graf M., Interlandi M., Moreno N., de Faria F.W., Kim S.N., Kastrati D., Korbanka S., Alfert A., Gerß J. (2020). Macrophage-tumor cell interaction promotes ATRT progression and chemoresistance. Acta Neuropathol..

[19] Stahl D., Knoll R., Gentles A.J., Vokuhl C., Buness A., Gütgemann I. (2021). Prognostic Gene Expression, Stemness and Immune Microenvironment in Pediatric Tumors. Cancers.

[20] Wang Y., Zhou C., Luo H., Cao J., Ma C., Cheng L., Yang Y. (2021). Prognostic implications of immune-related eight-gene signature in pediatric brain tumors. Braz. J. Med. Biol. Res..

[21] Grabovska Y., Mackay A., O’Hare P., Crosier S., Finetti M., Schwalbe E.C., Pickles J.C., Fairchild A.R., Avery A., Cockle J. (2020). Pediatric pan-central nervous system tumor analysis of immune-cell infiltration identifies correlates of antitumor immunity. Nat. Commun..

[22] Vermeulen J.F., Van Hecke W., Adriaansen E.J.M., Jansen M.K., Bouma R.G., Villacorta Hidalgo J., Fisch P., Broekhuizen R., Spliet W.G.M., Kool M. (2018). Prognostic relevance of tumor-infiltrating lymphocytes and immune checkpoints in pediatric medulloblastoma. Oncoimmunology.

[23] Quail D.F., Joyce J.A. (2017). The Microenvironmental Landscape of Brain Tumors. Cancer Cell.

[24] Tanabe S., Yamashita T. (2018). The role of immune cells in brain development and neurodevelopmental diseases. Int. Immunol..

[25] Morimoto K., Nakajima K. (2019). Role of the Immune System in the Development of the Central Nervous System. Front. Neurosci..

[26] Dunn G.P., Bruce A.T., Ikeda H., Old L.J., Schreiber R.D. (2002). Cancer immunoediting: From immunosurveillance to tumor escape. Nat. Immunol..

[27] Schreiber R.D., Old L.J., Smyth M.J. (2011). Cancer immunoediting: Integrating immunity’s roles in cancer suppression and promotion. Science.

[28] O’Sullivan T., Saddawi-Konefka R., Koebel W.V., Arthur C., White J.M., Uppaluri R., Andrews D.M., Ngiow S.F., Teng M.W.L., Smyth M.J. (2012). Cancer immunoediting by the innate immune system in the absence of adaptive immunity. J. Exp. Med..

[29] Simon A.K., Hollander G.A., McMichael A. (2015). Evolution of the immune system in humans from infancy to old age. Proc. R. Soc..

[30] Rieber N., Gille C., Köstlin N., Schäfer I., Spring B., Ost M., Spieles H., Kugel H.A., Pfeiffer M., Heininger V. (2013). Neutrophilic myeloid-derived suppressor cells in cord blood modulate innate and adaptive immune responses. Clin. Exp. Immunol..

[31] McGovern N., Shin A., Low G., Low D., Duan K., Yao L.J., Msallam R., Low I., Shadan N.B., Sumatoh H.R. (2017). Human fetal dendritic cells promote prenatal T-cell immune suppression through arginase-2. Nature.

[32] Olin A., Henckel E., Chen Y., Lakshmikanth T., Pou C., Mikes J., Gustafsson A., Bernhardsson A.K., Zhang C., Bohlin K. (2018). Stereotypic Immune System Development in Newborn Children. Cell.

[33] Kollmann T.R., Kampmann B., Mazmanian S.K., Marchant A., Levy O. (2017). Protecting the Newborn and Young Infant from Infectious Diseases: Lessons from Immune Ontogeny. Immunity.

[34] Engelhardt B., Vajkoczy P., Weller R.O. (2017). The movers and shapers in immune privilege of the CNS. Nat. Immunol..

[35] Vitkovic L., Konsman J.P., Bockaert J., Dantzer R., Homburger V., Jacque C. (2000). Cytokine signals propagate through the brain. Mol. Psychiatry.

[36] Warren K.E. (2018). Beyond the blood: Brain barrier: The importance of central nervous system (CNS) pharmacokinetics for the treatment of CNS tumors, including diffuse intrinsic pontine glioma. Front. Oncol..

[37] Phoenix T.N., Patmore D.M., Boop S., Boulos N., Jacus M.O., Patel Y.T., Roussel M.F., Finkelstein D., Goumnerova L., Perreault S. (2016). Medulloblastoma Genotype Dictates Blood Brain Barrier Phenotype. Cancer Cell.

[38] Ginn K.F., Gajjar A. (2012). Atypical teratoid rhabdoid tumor: Current therapy and future directions. Front. Oncol..

[39] Reddy A.T., Strother D.R., Judkins A.R., Burger P.C., Pollack I.F., Krailo M.D., Buxton A.B., Williams-Hughes C., Fouladi M., Mahajan A. (2020). Efficacy of High-Dose Chemotherapy and 3-D Conformal Radiation for Atypical Teratoid/Rhabdoid Tumor: A Report from the Children’s Oncology Group Trial ACNS0333. J. Clin. Oncol..

[40] Meel M.H., Guillén Navarro M., De Gooijer M.C., Metselaar D.S., Waranecki P., Breur M., Lagerweij T., Wedekind L.E., Koster J., Van De Wetering M.D. (2020). Mek/melk inhibition and blood-brain barrier deficiencies in atypical teratoid/rhabdoid tumors. Neuro-Oncology.

[41] Miron V.E., Boyd A., Zhao J.W., Yuen T.J., Ruckh J.M., Shadrach J.L., Van Wijngaarden P., Wagers A.J., Williams A., Franklin R.J.M. (2013). M2 microglia and macrophages drive oligodendrocyte differentiation during CNS remyelination. Nat. Neurosci..

[42] Bowman R.L., Klemm F., Akkari L., Pyonteck S.M., Sevenich L., Quail D.F., Dhara S., Simpson K., Gardner E.E., Iacobuzio-Donahue C.A. (2016). Macrophage Ontogeny Underlies Differences in Tumor-Specific Education in Brain Malignancies. Cell Rep..

[43] Mrdjen D., Pavlovic A., Hartmann F.J., Schreiner B., Utz S.G., Leung B.P., Lelios I., Heppner F.L., Kipnis J., Merkler D. (2018). High-Dimensional Single-Cell Mapping of Central Nervous System Immune Cells Reveals Distinct Myeloid Subsets in Health, Aging, and Disease. Immunity.

[44] Hammond T.R., Dufort C., Dissing-Olesen L., Giera S., Young A., Wysoker A., Walker A.J., Gergits F., Segel M., Nemesh J. (2019). Single-Cell RNA Sequencing of Microglia throughout the Mouse Lifespan and in the Injured Brain Reveals Complex Cell-State Changes. Immunity.

[45] Masuda T., Sankowski R., Staszewski O., Prinz M. (2020). Microglia Heterogeneity in the Single-Cell Era. Cell Rep..

[46] Jessa S., Blanchet-Cohen A., Krug B., Vladoiu M., Coutelier M., Faury D., Poreau B., De Jay N., Hébert S., Monlong J. (2019). Stalled developmental programs at the root of pediatric brain tumors. Nat. Genet..

[47] Li Q., Cheng Z., Zhou L., Darmanis S., Neff N.F., Okamoto J., Gulati G., Bennett M.L., Sun L.O., Clarke L.E. (2019). Developmental Heterogeneity of Microglia and Brain Myeloid Cells Revealed by Deep Single-Cell RNA Sequencing. Neuron.

[48] Svalina M.N., Kikuchi K., Abraham J., Lal S., Davare M.A., Settelmeyer T.P., Young M.C., Peckham J.L., Cho Y.J., Michalek J.E. (2016). IGF1R as a Key Target in High Risk, Metastatic Medulloblastoma. Sci. Rep..

[49] Yao M., Ventura P.B., Jiang Y., Rodriguez F.J., Wang L., Perry J.S.A., Yang Y., Wahl K., Crittenden R.B., Bennett M.L. (2020). Astrocytic trans-Differentiation Completes a Multicellular Paracrine Feedback Loop Required for Medulloblastoma Tumor Growth. Cell.

[50] Guadagno E., Presta I., Maisano D., Donato A., Pirrone C.K., Cardillo G., Corrado S.D., Mignogna C., Mancuso T., Donato G. (2018). Role of macrophages in brain tumor growth and progression. Int. J. Mol. Sci..

[51] Mohme M., Neidert M.C. (2020). Tumor-Specific T Cell Activation in Malignant Brain Tumors. Front. Immunol..

[52] Akkari L., Bowman R.L., Tessier J., Klemm F., Handgraaf S.M., de Groot M., Quail D.F., Tillard L., Gadiot J., Huse J.T. (2020). Dynamic changes in glioma macrophage populations after radiotherapy reveal CSF-1R inhibition as a strategy to overcome resistance. Sci. Transl. Med..

[53] Dang M.T., Gonzalez M.V., Gaonkar K.S., Rathi K.S., Young P., Arif S., Zhai L., Alam Z., Devalaraja S., To T.K.J. (2021). Macrophages in SHH subgroup medulloblastoma display dynamic heterogeneity that varies with treatment modality. Cell Rep..

[54] Crotty E.E., Smith S.M.C., Brasel K., Pakiam F., Girard E.J., Connor Y.D., Zindy F., Mhyre A.J., Roussel M.F., Olson J.M. (2021). Medulloblastoma recurrence and metastatic spread are independent of colony—Stimulating factor 1 receptor signaling and macrophage survival. J. Neurooncol..

[55] Yoshihara K., Shahmoradgoli M., Martínez E., Vegesna R., Kim H., Torres-Garcia W., Treviño V., Shen H., Laird P.W., Levine D.A. (2013). Inferring tumour purity and stromal and immune cell admixture from expression data. Nat. Commun..

[56] Newman A.M., Liu C.L., Green M.R., Gentles A.J., Feng W., Xu Y., Hoang C.D., Diehn M., Alizadeh A.A. (2015). Robust enumeration of cell subsets from tissue expression profiles. Nat. Methods.

[57] Becht E., Giraldo N.A., Lacroix L., Buttard B., Elarouci N., Petitprez F., Selves J., Laurent-Puig P., Sautès-Fridman C., Fridman W.H. (2016). Estimating the population abundance of tissue-infiltrating immune and stromal cell populations using gene expression. Genome Biol..

[58] Chakravarthy A., Furness A., Joshi K., Ghorani E., Ford K., Ward M.J., King E.V., Lechner M., Marafioti T., Quezada S.A. (2018). Pan-cancer deconvolution of tumour composition using DNA methylation. Nat. Commun..

[59] Jeschke J., Bizet M., Desmedt C., Calonne E., Dedeurwaerder S., Garaud S., Koch A., Larsimont D., Salgado R., Van Den Eynden G. (2017). DNA methylation-based immune response signature improves patient diagnosis in multiple cancers. J. Clin. Investig..

[60] Safaei S., Mohme M., Niesen J., Schüller U., Bockmayr M. (2021). DIMEimmune: Robust estimation of infiltrating lymphocytes in CNS tumors from DNA methylation profiles. Oncoimmunology.

[61] Wang Z., Guo X., Gao L., Wang Y., Guo Y., Xing B., Ma W. (2021). Classification of pediatric gliomas based on immunological profiling: Implications for immunotherapy strategies. Mol. Ther. Oncolytics.

[62] Bockmayr M., Klauschen F., Maire C.L., Rutkowski S., Westphal M., Lamszus K., Scheuller U., Mohme M. (2019). Immunologic Profiling of Mutational and Transcriptional Subgroups in Pediatric and Adult High-Grade Gliomas. Cancer Immunol. Res..

[63] Petralia F., Tignor N., Reva B., Koptyra M., Chowdhury S., Rykunov D., Krek A., Ma W., Zhu Y., Ji J. (2020). Integrated Proteogenomic Characterization across Major Histological Types of Pediatric Brain Cancer. Cell.

[64] Albert T.K., Interlandi M., Sill M., Graf M., Moreno N., Menck K., Rohlmann A., Melcher V., Korbanka S., Meyer zu Hörste G. (2021). An extracellular vesicle-related gene expression signature identifies high-risk patients in medulloblastoma. Neuro-Oncology.

[65] Plant A.S., Koyama S., Sinai C., Solomon I.H., Griffin G.K., Ligon K.L., Bandopadhayay P., Betensky R., Emerson R., Dranoff G. (2018). Immunophenotyping of pediatric brain tumors: Correlating immune infiltrate with histology, mutational load, and survival and assessing clonal T cell response. J. Neurooncol..

[66] Maximov V., Chen Z., Wei Y., Robinson M.H., Herting C.J., Shanmugam N.S., Rudneva V.A., Goldsmith K.C., MacDonald T.J., Northcott P.A. (2019). Tumour-associated macrophages exhibit anti-tumoural properties in Sonic Hedgehog medulloblastoma. Nat. Commun..

[67] Lu J.-Q., Wilson B.A., Yong V.W., Pugh J., Mehta V. (2012). Immune cell infiltrates in atypical teratoid/rhabdoid tumors. Can. J. Neurol. Sci. J. Can. Sci. Neurol..

[68] Marcu A., Schlosser A., Keupp A., Trautwein N., Johann P., Wölfl M., Lager J., Monoranu C.M., Walz J.S., Henkel L.M. (2021). Natural and cryptic peptides dominate the immunopeptidome of atypical teratoid rhabdoid tumors. J. Immunother. Cancer.

[69] Griesinger A.M., Birks D.K., Donson A.M., Amani V., Hoffman L.M., Waziri A., Wang M., Handler M.H., Foreman N.K. (2013). Characterization of Distinct Immunophenotypes across Pediatric Brain Tumor Types. J. Immunol..

[70] Teo W.Y., Elghetany M.T., Shen J., Man T.K., Li X., Chintagumpala M., Su J.M.F., Dauser R., Whitehead W., Adesina A.M. (2014). Therapeutic implications of CD1d expression and tumor-infiltrating macrophages in pediatric medulloblastomas. J. Neurooncol..

[71] Margol A.S., Robison N.J., Gnanachandran J., Hung L.T., Kennedy R.J., Vali M., Dhall G., Finlay J.L., Erdreich-Epstein A., Krieger M.D. (2015). Tumor-associated macrophages in SHH subgroup of medulloblastomas. Clin. Cancer Res..

[72] Pham C.D., Flores C., Yang C., Pinheiro E.M., Yearley J.H., Sayour E.J., Pei Y., Moore C., McLendon R.E., Huang J. (2016). Differential Immune Microenvironments and Response to Immune Checkpoint Blockade among Molecular Subtypes of Murine Medulloblastoma. Clin. Cancer Res..

[73] Aoki T., Hino M., Koh K., Kyushiki M., Kishimoto H., Arakawa Y., Hanada R., Kawashima H., Kurihara J., Shimojo N. (2016). Low Frequency of Programmed Death Ligand 1 Expression in Pediatric Cancers. Pediatr. Blood Cancer.

[74] Bockmayr M., Mohme M., Klauschen F., Winkler B., Budczies J., Rutkowski S., Schüller U. (2018). Subgroup-specific immune and stromal microenvironment in medulloblastoma. Oncoimmunology.

[75] Diao S., Gu C., Zhang H., Yu C. (2020). Immune cell infiltration and cytokine secretion analysis reveal a non-inflammatory microenvironment of medulloblastoma. Oncol. Lett..

[76] Witt D.A., Donson A.M., Amani V., Moreira D.C., Sanford B., Hoffman L.M., Handler M.H., Levy J.M.M., Jones K.L., Nellan A. (2018). Specific expression of PD-L1 in RELA-fusion supratentorial ependymoma: Implications for PD-1-targeted therapy. Pediatr. Blood Cancer.

[77] Martin A.M., Bell W.R., Yuan M., Harris L., Poore B., Arnold A., Engle E.L., Asnaghi L., Lim M., Raabe E.H. (2020). PD-L1 expression in pediatric low-grade gliomas is independent of BRAF V600E mutational status. J. Neuropathol. Exp. Neurol..

[78] Mackay A., Burford A., Molinari V., Jones D.T.W., Izquierdo E., Brouwer-Visser J., Giangaspero F., Haberler C., Pietsch T., Jacques T.S. (2018). Molecular, Pathological, Radiological, and Immune Profiling of Non-brainstem Pediatric High-Grade Glioma from the HERBY Phase II Randomized Trial. Cancer Cell.

[79] Panwalkar P., Pratt D., Chung C., Dang D., Le P., Martinez D., Bayliss J.M., Smith K.S., Adam M., Potter S. (2020). SWI/SNF complex heterogeneity is related to polyphenotypic differentiation, prognosis, and immune response in rhabdoid tumors. Neuro-Oncology.

[80] Alfert A., Moreno N., Kerl K. (2019). The BAF complex in development and disease. Epigenet. Chromatin.

[81] Northcott P.A., Pfister S.M., Jones D.T.W. (2015). Next-generation (epi)genetic drivers of childhood brain tumours and the outlook for targeted therapies. Lancet Oncol..

[82] Pan D., Kobayashi A., Jiang P., De Andrade L.F., Tay R.E., Luoma A.M., Tsoucas D., Qiu X., Lim K., Rao P. (2018). A major chromatin regulator determines resistance of tumor cells to T cell–mediated killing. Science.

[83] Biegel J.A., Zhou J.Y., Rorke L.B., Stenstrom C., Wainwright L.M., Fogelgren B. (1999). Germ-line and acquired mutations of INI1 in atypical teratoid and rhabdoid tumors. Cancer Res..

[84] Hasselblatt M., Nagel I., Oyen F., Bartelheim K., Russell R.B., Schüller U., Junckerstorff R., Rosenblum M., Alassiri A.H., Rossi S. (2014). SMARCA4-mutated atypical teratoid/rhabdoid tumors are associated with inherited germline alterations and poor prognosis. Acta Neuropathol..

[85] Price G., Bouras A., Hambardzumyan D., Hadjipanayis C.G. (2021). Current knowledge on the immune microenvironment and emerging immunotherapies in diffuse midline glioma. EBioMedicine.

[86] Johann P.D., Erkek S., Zapatka M., Hasselblatt M., Pfister S.M., Kool M. (2016). Atypical Teratoid/Rhabdoid Tumors Are Comprised of Three Epigenetic Subgroups with Distinct Enhancer Landscapes. Cancer Cell.

[87] Luke J.J., Bao R., Sweis R.F., Spranger S., Gajewski T.F. (2019). WNT/b-catenin pathway activation correlates with immune exclusion across human cancers. Clin. Cancer Res..

[88] Grund-Gröschke S., Stockmaier G., Aberger F. (2019). Hedgehog/GLI signaling in tumor immunity—New therapeutic opportunities and clinical implications. Cell Commun. Signal..

[89] Mehlman C., Kamga P.T., Costantini A., Julié C., Dumenil C., Dumoulin J., Ouaknine J., Giraud V., Chinet T., Emile J.F. (2021). Baseline hedgehog pathway activation and increase of plasma Wnt1 protein are associated with resistance to immune checkpoint inhibitors in advanced non-small-cell lung cancer. Cancers.

[90] Sevenich L. (2019). Turning “Cold” into “Hot” tumors—Opportunities and challenges for radio-immunotherapy against primary and metastatic brain cancers. Front. Oncol..

[91] Galluzzi L., Humeau J., Buqué A., Zitvogel L., Kroemer G. (2020). Immunostimulation with chemotherapy in the era of immune checkpoint inhibitors. Nat. Rev. Clin. Oncol..

[92] Karachi A., Dastmalchi F., Mitchell D.A., Rahman M. (2018). Temozolomide for immunomodulation in the treatment of glioblastoma. Neuro-Oncology.

[93] Petroni G., Buqué A., Zitvogel L., Kroemer G., Galluzzi L. (2021). Immunomodulation by targeted anticancer agents. Cancer Cell.

[94] Huang S., Wang Z., Zhou J., Huang J., Zhou L., Luo J., Wan Y.Y., Long H., Zhu B. (2019). EZH2 inhibitor GSK126 suppresses antitumor immunity by driving production of myeloid-derived suppressor cells. Cancer Res..

[95] Alimova I., Birks D.K., Harris P.S., Knipstein J.A., Venkataraman S., Marquez V.E., Foreman N.K., Vibhakar R. (2013). Inhibition of EZH2 suppresses self-renewal and induces radiation sensitivity in atypical rhabdoid teratoid tumor cells. Neuro-Oncology.

[96] Knutson S.K., Warholic N.M., Wigle T.J., Klaus C.R., Allain C.J., Raimondi A., Scott M.P., Chesworth R., Moyer M.P., Copeland R.A. (2013). Durable tumor regression in genetically altered malignant rhabdoid tumors by inhibition of methyltransferase EZH2. Proc. Natl. Acad. Sci. USA.

[97] Chi S.N., Bourdeaut F., Laetsch T.W., Fouladi M., Macy M.E., Makin G.W.J., Shukla N.N., Wetmore C., Margol A.S., Casanova M. (2020). Phase I study of tazemetostat, an enhancer of zeste homolog-2 inhibitor, in pediatric pts with relapsed/refractory integrase interactor 1-negative tumors. J. Clin. Oncol..

[98] Jones R.L., Blay J., Agulnik M., Chugh R., Mir O., Italiano A., Thomas D., Gupta A., Jahan T., Cote G. (2018). A phase 2, multicenter study of the EZH2 inhibitor tazemetostat in adults (rhabdoid tumor cohort) (NCT02601950). Ann. Oncol..

[99] Gounder M., Schöffski P., Jones R.L., Agulnik M., Cote G.M., Villalobos V.M., Attia S., Chugh R., Chen T.W.W., Jahan T. (2020). Tazemetostat in advanced epithelioid sarcoma with loss of INI1/SMARCB1: An international, open-label, phase 2 basket study. Lancet Oncol..

[100] Keane L., Cheray M., Saidi D., Kirby C., Friess L., Gonzalez-rodriguez P., Gerdes M.E., Grabert K., McColl B.W. (2021). Inhibition of microglial EZH2 leads to anti-tumoral effects in pediatric diffuse midline gliomas. Neuro-Oncology.

[101] Woods D.M., Sodré A.L., Villagra A., Sarnaik A., Sotomayor E.M., Weber J. (2015). HDAC inhibition upregulates PD-1 ligands in melanoma and augments immunotherapy with PD-1 blockade. Cancer Immunol. Res..

[102] Knox T., Sahakian E., Banik D., Hadley M., Palmer E., Noonepalle S., Kim J., Powers J., Gracia-Hernandez M., Oliveira V. (2019). Selective HDAC6 inhibitors improve anti-PD-1 immune checkpoint blockade therapy by decreasing the anti-inflammatory phenotype of macrophages and down-regulation of immunosuppressive proteins in tumor cells. Sci. Rep..

[103] Sharma P., Hu-Lieskovan S., Wargo J.A., Ribas A. (2017). Primary, Adaptive, and Acquired Resistance to Cancer Immunotherapy. Cell.

[104] Ring E.K., Markert J.M., Gillespie G.Y., Friedman G.K. (2017). Checkpoint proteins in pediatric brain and extracranial solid tumors: Opportunities for immunotherapy. Clin. Cancer Res..

[105] Majzner R.G., Simon J.S., Grosso J.F., Martinez D., Pawel B.R., Santi M., Merchant M.S., Geoerger B., Hezam I., Marty V. (2017). Assessment of programmed death-ligand 1 expression and tumor-associated immune cells in pediatric cancer tissues. Cancer.

[106] Haydar D., Houke H., Chiang J., Yi Z., Odé Z., Caldwell K., Zhu X., Mercer K.S., Stripay J.L., Shaw T.I. (2021). Cell-surface antigen profiling of pediatric brain tumors: B7-H3 is consistently expressed and can be targeted via local or systemic CAR T-cell delivery. Neuro-Oncology.

[107] Majzner R.G., Theruvath J.L., Nellan A., Heitzeneder S., Cui Y., Mount C.W., Rietberg S.P., Linde M.H., Xu P., Rota C. (2019). CAR T cells targeting B7-H3, a pan-cancer antigen, demonstrate potent preclinical activity against pediatric solid tumors and brain tumors. Clin. Cancer Res..

[108] Theruvath J., Sotillo E., Mount C.W., Graef C.M., Delaidelli A., Heitzeneder S., Labanieh L., Dhingra S., Leruste A., Majzner R.G. (2020). Locoregionally administered B7-H3-targeted CAR T cells for treatment of atypical teratoid/rhabdoid tumors. Nat. Med..

[109] Donovan L.K., Delaidelli A., Joseph S.K., Bielamowicz K., Fousek K., Holgado B.L., Manno A., Srikanthan D., Gad A.Z., Van Ommeren R. (2020). Locoregional delivery of CAR T cells to the cerebrospinal fluid for treatment of metastatic medulloblastoma and ependymoma. Nat. Med..

[110] Gupta A., Taslim C., Tullius B.P., Cripe T.P. (2020). Therapeutic modulation of the CD47-SIRPα axis in the pediatric tumor microenvironment: Working up an appetite. Cancer Drug Resist..

[111] Gholamin S., Mitra S.S., Feroze A.H., Liu J., Kahn S.A., Zhang M., Esparza R., Richard C., Ramaswamy V., Remke M. (2017). Disrupting the CD47-SIRPα anti-phagocytic axis by a humanized anti-CD47 antibody is an efficacious treatment for malignant pediatric brain tumors. Sci. Transl. Med..

[112] Ravanpay A.C., Gust J., Johnson A.J., Rolczynski L.S., Chang C.A., Hoglund V.J., Mukherjee R., Nicholas A., Orentas R.J., Jensen M.C. (2019). EGFR806-CAR T cells selectively target a tumor-restricted EGFR epitope in glioblastoma. Oncotraget.

[113] Stastny M.J., Brown C.E., Ruel C., Jensen M.C. (2007). Medulloblastomas Expressing IL13Ra2 are Targets for IL13-zetakine+ Cytolytic T Cells. J. Pediatr. Hematol. Oncol..

[114] Ahmed N., Ratnayake M., Savoldo B., Perlaky L., Dotti G., Wels W.S., Bhattacharjee M.B., Gilbertson R.J., Shine H.D., Weiss H.L. (2007). Regression of Experimental Medulloblastoma following Transfer of HER2-Specific T Cells. Cancer Res..

[115] Nellan A., Rota C., Majzner R., Lester-McCully C.M., Griesinger A.M., Mulcahy Levy J.M., Foreman N.K., Warren K.E., Lee D.W. (2018). Durable regression of Medulloblastoma after regional and intravenous delivery of anti-HER2 chimeric antigen receptor T cells. J. Immunother. Cancer.

[116] Liu D., Song L., Brawley V.S., Robison N., Wei J., Gao X., Tian G., Margol A., Ahmed N., Asgharzadeh S. (2013). Medulloblastoma expresses CD1d and can be targeted for immunotherapy with NKT cells. Clin. Immunol..

[117] Orlando D., Miele E., De Angelis B., Guercio M., Boffa I., Sinibaldi M., Po A., Caruana I., Abballe L., Carai A. (2018). Adoptive immunotherapy using PRAME-specific T cells in medulloblastoma. Cancer Res..

[118] Powell A.B., Yadavilli S., Saunders D., Van Pelt S., Chorvinsky E., Burga R.A., Albihani S., Hanley P.J., Xu Z., Pei Y. (2019). Medulloblastoma rendered susceptible to NK-cell attack by TGFβ neutralization. J. Transl. Med..

[119] Tan I., Arifa R.D.N., Rallapalli H., Kana V., Lao Z., Sanghrajka R.M., Bayin N.S., Tanne A., Wojcinski A., Korshunov A. (2021). CSF1R Inhibition Depletes Tumor-Associated Macrophages and Attenuates Tumor Progression in a Mouse Sonic Hedgehog- Medulloblastoma Model. Oncogene.

[120] Mount C.W., Majzner R.G., Sundaresh S., Arnold E.P., Kadapakkam M., Haile S., Labanieh L., Hulleman E., Woo P.J., Rietberg S.P. (2018). Potent antitumor efficacy of anti-GD2 CAR T cells in H3-K27M(+) diffuse midline gliomas. Nat. Med..

[121] van Gool S.W., Holm S., Rachor J., Adamson L., Technau A., Maass E., Frühwald M.C., Schlegel P.G., Eyrich M. (2016). Immunotherapy in atypical teratoid-rhabdoid tumors: Data from a survey of the HGG-Immuno Group. Cytotherapy.

[122] Caruso D.A., Orme L.M., Neale A.M., Radcliff F.J., Armor G.M., Maixner W., Downie P., Hassall T.E., Tang M.L.K., Ashley D.M. (2004). Results of a phase 1 study utilizing monocyte-derived dendritic cells pulsed with tumor RNA in children and young adults with brain cancer. Neuro-Oncology.

[123] Ardon H., De Vleeschouwer S., Claes L., Van Calenbergh F., Kramm C.M., Rutkowski S., Wolff J.E.A., Van Gool S.W. (2010). Adjuvant Dendritic Cell-Based Tumour Vaccination for Children With Malignant Brain Tumours. Pediatr. Blood Cancer.

[124] Garcia-Moure M., Gonzalez-Huarriz M., Labiano S., Guruceaga E., Bandres E., Zalacain M., Marrodan L., de Andrea C., Villalba M., Martinez-Velez N. (2021). Delta-24-RGD, an oncolytic adenovirus, increases survival and promotes proinflammatory immune landscape remodeling in models of AT/RT and CNS-PNET. Clin. Cancer Res..

[125] Bonifant C.L., Jackson H.J., Brentjens R.J., Curran K.J. (2016). Toxicity and management in CAR T-cell therapy. Mol. Ther. Oncolytics.

